# Innovative Utilization of Citrus Sinensis Peel Hydrogels: Enhancing Soil Water Retention and Efficient Removal of Methylene Blue from Wastewater

**DOI:** 10.3390/polym17030428

**Published:** 2025-02-06

**Authors:** Bingqin Teng, Jun Wu, Yuan Zhong, Yinhua Wang, Decheng Qiao, Runqi Quan, Zhengqian Zhou, Liqun Cai, Peng Qi, Zhuzhu Luo, Xiaodong Zhang

**Affiliations:** 1 College of Resources and Environment, Gansu Agricultural University, Lanzhou 730070, China; bingqinteng@outlook.com (B.T.); ww19944159607@outlook.com (Y.W.); 18706986255@163.com (D.Q.); quanrunqi@163.com (R.Q.); 18294897803@163.com (Z.Z.); cailq@gsau.edu.cn (L.C.); qipeng0714@emails.bjut.edu.cn (P.Q.); luozz@gsau.edu.cn (Z.L.); m18409492603@163.com (X.Z.); 2 State Key Laboratory of Aridland Crop Science, Gansu Agricultural University, Lanzhou 730070, China; zhongy@gsau.edu.cn; 3 China Gansu Water-Saving Agricultural Engineering and Technology Research Center, Lanzhou 730070, China

**Keywords:** Citrus sinensis peel hydrogel, superabsorbent hydrogel, soil water retention, methylene blue adsorption, wastewater treatment

## Abstract

In the context of increasing water scarcity and environmental pollution, this study investigates the synthesis and application of p(AA-Oco-AAm)-g-Citrus Sinensis Peel hydrogel (CSP hydrogel) to enhance soil water retention and remove organic dyes from wastewater. Hydrogels were prepared using a combination of acrylamide and acrylic acid, with the incorporation of citrus peel as a natural resource. The water absorption capacity of the hydrogels was evaluated, achieving a maximum retention rate of 477 g/g, significantly improving the water-holding ability of various soil types. Additionally, the hydrogels demonstrated a strong affinity for methylene blue, with an equilibrium adsorption capacity reaching 2299.45 mg/g, indicating their effectiveness in wastewater treatment. Kinetic and isothermal adsorption models were applied to analyze the adsorption dynamics, revealing a superior fit to the Langmuir model. The hydrogels maintained structural integrity and reusability over multiple cycles, underscoring their potential for sustainable agricultural practices and environmental remediation. This research highlights the dual benefits of utilizing agricultural waste for the development of eco-friendly materials while addressing critical challenges in water management and pollution control.

## 1. Introduction

In the context of global climate change and population growth, drought and water scarcity have become crucial problems affecting agricultural production and ecological environments [1]. As water is an essential element for plant growth, the water-retaining capacity of soil is directly related to crop growth and yield. Enhancing the water-retention ability of soil can not only improve crop drought resistance but also promote the efficient use of water resources and the development of sustainable agriculture [2,3]. Thus, finding efficient soil amendments to improve the water-retention performance of soil has become an important direction in agricultural research. With global industrialization advancing at an accelerated pace, water pollution has also become an increasingly serious environmental issue [4,5,6]. Industrial discharges, particularly those containing dyes, have had a significant impact on aquatic ecosystems [7,8,9]. These dyes, especially synthetic ones like methylene blue, are difficult to degrade and pose threats to both aquatic life and human health, necessitating the development of effective water treatment methods. Biopolymer-based hydrogels stand out for their superior adsorptive properties and environmental friendliness, making them ideal materials for the removal of these harmful substances [10,11,12]. Research into hydrogels and their applications, especially in water treatment, holds significant theoretical and practical value. The use of natural or modified materials to create hydrogels not only effectively removes toxic compounds from water but also avoids secondary contamination associated with traditional methods [10,13,14,15]. Furthermore, the utilization of agricultural waste in the production of hydrogels aligns with sustainable development principles, contributing to resource recycling and environmental protection [16,17].

In recent years, superabsorbent hydrogels (SAHs) have received extensive attention due to their excellent water-adsorption and-retention capabilities [16]. Emerging evidence suggests that hydrogels can enhance the water-retention capacity of soil, particularly in drought-prone environments, which may contribute to improved plant growth conditions [16,18]. Hydrogels based on polyacrylamide (AAM) and polyacrylic acid (AA) exhibit excellent hydrophilicity and water-retention properties, making them ideal materials for the enhancement of the water-retention capacity of soil [19,20]. In addition, using agricultural waste (such as citrus peel) as raw materials for hydrogel synthesis can not only reduce production costs but also realize resource recycling, which has important ecological and economic value [21]. To date, numerous studies have focused on the application of hydrogels in dye removal, especially on the adsorption properties of organic dyes [22,23]. According to the existing literature, researchers have extensively explored various hydrogels based on natural and synthetic polymers and their effects in wastewater treatment [24,25]. Numerous studies have demonstrated that synthetic hydrogels, particularly polyacrylic acid (PAA) and polyvinyl alcohol (PVA), exhibit significant adsorption capabilities in removing a variety of dye pollutants from aqueous solutions. For instance, PVA-based hydrogels have proven effective in adsorbing aniline dyes, with removal efficiencies reaching up to 88% for Congo red and 92% for Basic Blue 3 [26]. Additionally, PAA hydrogels have been shown to remove over 95% of methylene blue within 30 min [27]. These high removal rates are attributed to the abundance of hydrophilic groups (-COOH and -OH) in these polymers, which facilitate strong interactions with dye molecules through hydrogen bonding and electrostatic attraction. Furthermore, the porous structure of hydrogels provides a large surface area for adsorption to occur. Meanwhile, hydrogels prepared from natural and modified materials also display remarkable adsorption properties. Hydrogels based on plants, seaweed, fruit peels, etc., have attracted wide attention due to their abundance of hydrophilic groups and large surface areas [28,29,30]. Studies on these materials have demonstrated that, compared with traditional water treatment technologies, hydrogels can not only remove water pollutants more efficiently but also show the advantages of low cost and environmental friendliness in application. Regarding the adsorption mechanism, previous studies have revealed multiple influencing factors, such as contact time, temperature, pH value, and initial concentration [31,32,33]. These factors have a significant impact on the adsorption properties of hydrogels. Therefore, when studying the applicability of hydrogels, it is necessary to deeply analyze the interaction between them and the target pollutants. Some research on hybrid hydrogels has also been carried out in recent years. By combining multiple materials, the mechanical and adsorption properties of hydrogels can be improved [34,35,36]. This innovative method provides broader options for water treatment and shows the potential for multifunctionality. In the research field of hydrogels, there have been many explorations regarding the application of hydrogels in pollutant removal, and the theoretical basis and experimental research are relatively sufficient. However, it is still necessary to enhance the water retention performance of hydrogels on soil with different textures, the adsorption of specific dyes (such as methylene blue), and the synthesis of hydrogels from agricultural organic waste. Plant-based hydrogels generally exhibit higher Q_m values (e.g., 2967.6 mg/g for starch-based hydrogel) compared to synthetic P(AA-AAm) hydrogels (1950 mg/g). This is attributed to the natural porosity and diversity of functional groups in plant-derived polymers [37].

This study focuses on the utilization of (AA-co-AAM) citrus-peel hydrogels to improve the water-retention ability of soil and adsorb methylene blue dye. Citrus peel is not only a common form of agricultural waste but is also rich in functional components such as natural polysaccharides, and its extract provides abundant raw materials for hydrogel synthesis. Through an efficient synthesis process, the prepared hydrogels are expected to show good adsorption ability, and optimizing the synthesis conditions can further improve their water-adsorption effect and methylene blue removal efficiency. This study aims to provide a comprehensive understanding of the adsorption mechanism and properties of (AA-co-AAM) citrus-peel hydrogels through a series of performance tests such as the use of adsorption kinetics and isothermal adsorption model fitting. This study not only provides new ideas for agricultural drought management but also offers a new technical route for sewage treatment, which is of great practical significance for the achievement of the sustainable development of agriculture and the environment.

## 2. Materials and Methods

### 2.1. Experimental Materials

All the chemical reagents used in this study—namely, ammonium persulfate (APS), acrylamide (AAm), N,N’-methylene bisacrylamide (MBA), methylene blue (MB), acrylic acid (AA), and sodium hydroxide (NaOH)—were purchased from Shanghai Macklin Biochemical Co., Ltd. (Shanghai, China), with a purity of analytical grade.

### 2.2. Preparation of p(AA-co-AAm)-g-Citrus Sinensis Peel Hydrogel (CSP Hydrogel)

**Figure 1:**
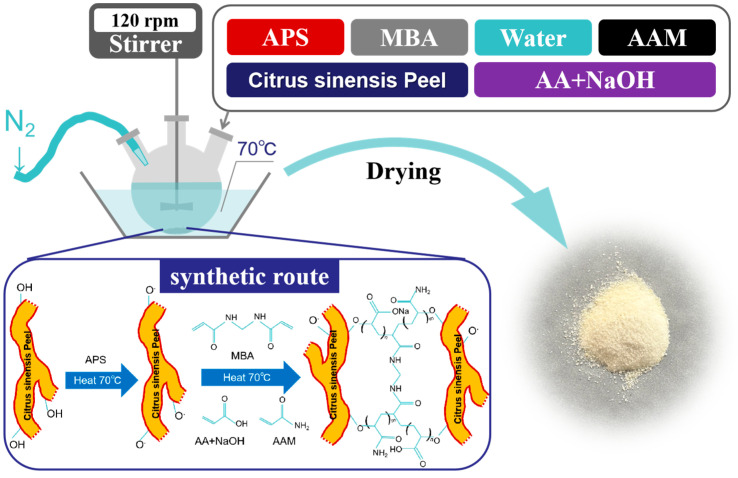
Route map for CSP hydrogel synthesis. In the figure, APS denotes ammonium persulfate, AA denotes acrylic acid, AAM denotes acrylamide, and MBA denotes N,N’-methylene bisacrylamide.

The Citrus sinensis peel used in this study was produced in Gannan, Jiangxi, China. The total weight of the hydrogel prepared in this study was 500 g. The synthesis steps of p(AA-co-AAm)-g-citrus sinensis peel hydrogel (CSP hydrogel) are outlined as follows:

First, a quantified and fully crushed Citrus sinensis peel (CSP) was mixed with deionized water (300 mL, 60 wt%). Next, the suspension was transferred into a three-necked flask, purged with N_2_ for 30 min, and simultaneously heated in a water bath to 70 °C. Then, a quantified amount of ammonium persulfate (APS) was added, and the mixture was stirred for 30 min. Subsequently, quantified amounts of acrylamide (AAm), acrylic acid (AA neutralized with NaOH to 40%), N,N’-methylenebisacrylamide (MBA), a cross-linker (Table 1), and the remaining deionized water (149 mL, 29.8 wt%) were added. Polymerization was conducted at 70 °C in a water bath. Gelation occurred after approximately 15 min of heating, and post-gelation polymerization continued for an additional 24 h to ensure complete cross-linking. After gel formation, the gel was washed three times with ethanol to remove unreacted monomers, then washed three more times with ultrapure water to remove the residual ethanol. Finally, the sample was dried in an oven at 40 °C, and the dried hydrogel was pulverized and sieved (0.5 mm) [36]. A schematic diagram of the CSP hydrogel synthesis route is shown in Figure 1.

Regarding the potential toxicity of polyacrylic acid and polyacrylamide hydrogels in water bodies, according to existing research and data, these two materials are generally considered to be biocompatible and non-toxic. Acrylamide monomer is considered toxic and carcinogenic in some cases. However, in hydrogels, acrylamide usually exists in a polymerized form, and a small amount of unreacted acrylamide monomer has been removed by ethanol washing, so its toxicity can be greatly reduced [38].

### 2.3. Characterization Tests of CSP Hydrogel

The FT-IR spectra of the samples were measured in the 400–4000 cm^−1^ wavelength range with an FT-IR spectral analyzer (Thermo Fisher Nicolet iS50, Thermo Fisher Scientific, Waltham, MA, USA). The powders were rapidly pressed at 10–15 MPa for this measurement. The surface morphology of the samples was observed by spraying gold under a scanning electron microscope (SEM) (JEOL S-3400N, HITACHI, Shenzhen, China). The SEM had a thickness of 53 mm and a voltage of 10 kV. Thermogravimetric analysis (TGA) of the samples was carried out in a nitrogen atmosphere using a Mettler TG-DSC 3+ thermogravimetric analyzer from METTLER TOLEDO (Zurich, Switzerland). The temperature range was 25–900 °C, and the ramp rate was 10 °C per minute.

### 2.4. CSP Hydrogel Water-Absorption Test

#### 2.4.1. Plotting of the Water-Absorption Curve

A certain mass of CSP hydrogel was placed into a tea bag. Then, the tea bag was immersed in ultrapure water, and the amount of water adsorbed (Water Q) at different times was measured. The ultimately obtained water-absorption curve was fitted using the Langmuir model to obtain key parameters such as the fitting coefficient (R2), the maximum absorption capacity (q), and the absorption rate (k). The formula is expressed as follows:

Water Q = (W − W0)/W0(1)

Langmuir model: y = q × x/(k + x)(2)

where W is the weight of the sample after water absorption and W0 is the weight of the dry sample.

#### 2.4.2. Durability Test

The durability of CSP hydrogel was evaluated by measuring the swelling capacity of CSP-added hydrogel and control hydrogel during the repeated water-absorption and drying processes. Specifically, 0.05 g of hydrogel encapsulated in a tea bag was immersed in ultrapure water for 24 h; then, the tea bag was taken out and weighed as the equilibrium weight. The measured water-absorption amount was regarded as its equilibrium absorption amount (Water Qeq). The hydrogel was placed in an oven (50 °C) and dried until a constant weight was reached. Then, the tea bag was taken out and weighed again. This process was repeated 8 times. The specific formula is expressed as follows:

Water Qeq = (Weq − W0)/W0(3)

where Weq is the weight of the water-absorbed sample after reaching equilibrium and W0 is the weight of the dry sample.

#### 2.4.3. Water Absorption in Different pH Solutions

A certain mass of CSP hydrogel was added to aqueous solutions with pH values of 3, 4, 5, 6, 7, 8, 9, 10, 11, and 12. The equilibrium absorption amounts (Water Qeq) of the aqueous solutions with different pH values were measured. Then, the absorption amount (Water Qeq) of CSP hydrogel in different pH solutions was fitted using the Gaussian distribution model.

(4) $$\mathrm{Gaussian}\;\mathrm{distribution}\;\mathrm{function}:\;y=a\cdot e^{-\frac{{\left(x-b\right)}^{2}}{{2c}^{2}}}$$

where *a*, *b*, and *c* are all function coefficients; *a* is the height of the peak, also known as the amplitude; *b* is the mean value, representing the central position of the distribution; and *c* is the standard deviation, controlling the width of the curve.

#### 2.4.4. Influence of CSP Hydrogel Addition on Soils with Different Textures

Since the soil in the northwest region is mostly sandy soil and loam soil, the distribution of organic matter is uneven, and there are also large quantities of saline–alkali soils. Therefore, the soils used in this study are of four types: loam, sand, organic loam, and saline sand. CSP hydrogels with mass ratios of 0.1%, 0.3%, 0.5%, and 0.7% were added to flowerpots containing 100 g of soil with different textures (see the Table 2). After soaking the bottom of the flowerpots in tap water for one day, the soil moisture content was measured. Then, the flowerpots were placed in an environment with a temperature of 20 ± 0.5 °C and a humidity of 20% ± 0.5%. The soil moisture content was measured every day until the soil moisture content was 0%. The soil moisture content curve was obtained. The formula is expressed as follows:

Soil moisture content (ω) = Mw/Ms(5)

where Mw is the weight of water in the soil and Ms is the dry weight of the soil.

### 2.5. Characterization of CSP Hydrogel via Methylene Blue (MB) Adsorption and Desorption Experiments

**Figure 2:**
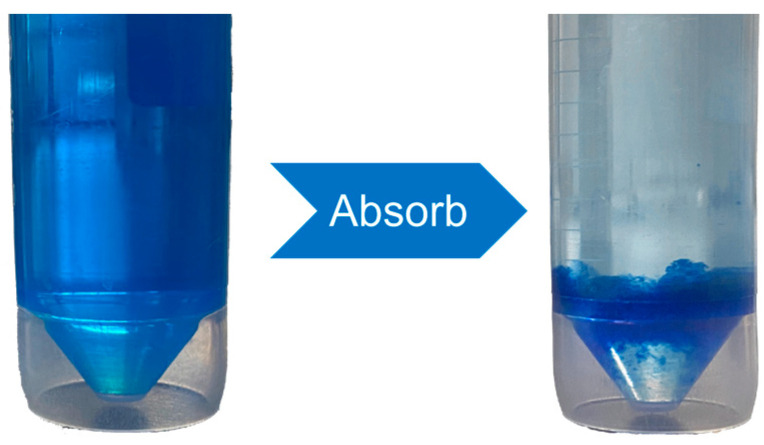
Schematic illustration of the adsorption of methylene blue (MB) by CSP hydrogels.

First, 10 mg of CSP hydrogels with different treatments were added to 25 mL of MB solution (initial concentration of Ce = 480 mg/L and intrinsic pH of 7.0 ± 0.3). Each treatment was repeated three times. After 12 h of agitation (180 rpm, 25 °C), the supernatant was diluted and analyzed at 660 nm using a UV spectrophotometer (SP-756P) (For desorption studies see Section 2.5.4). The morphology of the hydrogel both before and after MB adsorption is shown in Figure 2. The group of materials exhibiting the best adsorption of MB were selected as the materials for subsequent tests.

#### 2.5.1. Effects of Different CSP Hydrogel Dosages on MB Adsorption

Various amounts of CSP hydrogels (2, 4, 6, 8, 10, 12, 14, 16, 18, and 20 mg) were added to 25 mL of MB solution with an initial concentration (Ce) of 480 mg/L. The mixtures were agitated at 25 °C for 12 h, and the equilibrium adsorption capacity (Qeq) and MB removal rate (R) were determined. The formulas used for these calculations are expressed as follows:

Qeq = (Ce − Ceq) V/m(6)

R = (1 − Ceq/Ce) · 100%(7)

where Ceq represents the equilibrium concentration of MB adsorbed by the hydrogel, Ce is the initial MB concentration, V is the volume of the MB solution, and m is the mass of hydrogel added.

#### 2.5.2. Isothermal Adsorption Studies

The equilibrium adsorption capacity (Qeq) of 5 mg CSP hydrogels was measured in 25 mL of MB solution with concentrations (Ce) ranging from 5 to 2000 mg/L at temperatures of 30, 25, and 20 °C after 12 h of agitation. The obtained data were fitted to Langmuir and Freundlich models to describe the adsorption isotherms. The equations for these models are expressed as follows:

Langmuir model: y = q × x/(k + x)(8)

Freundlich model: y = k × x^n^ (9)

where q is the maximum adsorption capacity, k is a parameter related to the adsorption energy, and n is a dimensionless constant indicating the favorability of the adsorption process.

#### 2.5.3. Kinetics of MB Adsorption by CSP Hydrogels

The adsorption kinetics of 100 mg CSP hydrogels were investigated by directly adding them to 500 mL of MB solution with initial concentrations (Ce) of 80, 160, and 240 mg/L (pH = 7.0 ± 0.3). Sampling was performed at various intervals up to 24 h, and the amount of absorbed MB (Qt) was measured. Additionally, the hydrogels were packed in tea bags and subjected to the same conditions for kinetic analysis. Pseudo first- and second-order models of adsorption kinetics were used to fit the experimental data. The formulas for these models are expressed as follows:

Pseudo-1st order: Qt = Qeq (1 − e − k1t)(10)

Pseudo-2nd order: 1/Qt = 1/(k2Qeq2) + t/Qeq(11)

where k1 and k2 are the rate constants for the adsorption processes and t is time.

#### 2.5.4. Desorption Studies

Desorption experiments were carried out by placing 20 mg CSP hydrogels in 100 mL of MB solution with concentrations (Ce) of 240 and 480 mg/L. MB-laden hydrogels were rinsed with deionized water (pH 7.0) and subsequently immersed in 0.1 M HCl (pH 1.5) under identical agitation conditions. Then, the hydrogels were filtered, and the desorption process was repeated five times to obtain the Qeq changes over successive desorption cycles.

#### 2.5.5. Effect of pH on the Adsorption of MB by CSP Hydrogels

The impact of pH on the adsorption of MB by CSP hydrogels was evaluated by introducing 5 mg hydrogels into 25 mL of MB solutions with concentrations (C0) of 80 mg/L at pH values ranging from 2 to 12. After 12 h of agitation at 25 °C, the equilibrium adsorption capacities were determined and analyzed for significant differences across the pH range.

### 2.6. Data Analysis

Data were collated in Microsoft Excel 2019. Statistical significance tests were conducted in SPSS 23.0. Graphs were generated using Excel 2019, PowerPoint 2019, and Origin 2021.

## 3. Results

### 3.1. Scanning Electron Microscopy (SEM)

**Figure 3:**
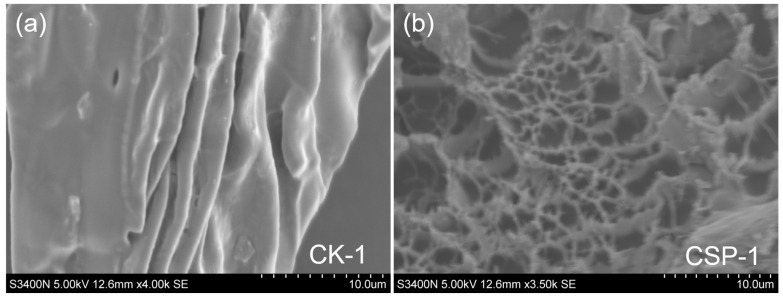
Hydrogel without Citrus sinensis peel addition (**a**); p(AA-co-AAm)-g-citrus sinensis peel hydrogel (**b**).

As clearly shown in Figure 3a, the surface of the hydrogel without CSP addition was relatively smooth, with only slight undulations and few wrinkles. In contrast, as presented in Figure 3b, the CSP hydrogel possessed a greater number of pores. The reticular structure of the CSP hydrogel enables it to maintain a certain structural stability in water and preserve its own structure after multiple uses. Although the hydrogel without CSP addition exhibits similar absorption characteristics, its structure is relatively simple, and its stability is inferior to that of the CSP hydrogel. Due to its smooth surface, it has a smaller specific surface area and fewer absorption sites compared to the CSP hydrogel. The enhanced porosity of the CSP hydrogel originates from the synergistic interaction between CSP and AA. CSP, with its branched polysaccharide structure, acts as a pore-forming template during cross-linking. Hydrogen bonding between CSP’s hydroxyl groups and AA’s carboxyl moieties induces localized phase separation, forming interconnected pores upon hydration. In contrast, hydrogels without CSP rely solely on AA-derived cross-links, which yield a denser, less porous architecture due to unidirectional chain growth. Consequently, CSP not only amplifies pore formation but also stabilizes the 3D network, improving structural resilience during repeated hydration cycles.

### 3.2. Infrared Spectral Analysis

**Figure 4:**
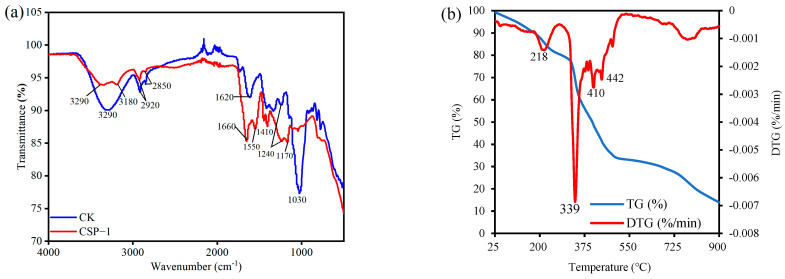
(**a**) Infrared spectra of CK (Citrus sinensis peel) and CSP-1 (CSP hydrogel); (**b**) thermogravimetric analysis of CSP hydrogel. In the figure, CSP refers to Citrus sinensis peel.

Figure 4a presents the infrared spectral curves of Citrus sinensis peel and CSP. In the figure, the ordinate represents transmittance. A lower transmittance implies a greater number of functional groups. In the infrared spectrum of CSP, there is a relatively wide band at 3290 cm^−1^, which is attributed to the −OH functional group. The band at 1030 cm^−1^ corresponds to the glycosidic bond. However, the peak at 1030 cm^−1^ in the CSP hydrogel disappears, indicating that during the synthesis of CSP, the glycosidic bonds are mainly broken and form C-O-C asymmetric oxygen bridges (at 1170 cm^−1^) in the CSP hydrogel. The formation of C-O-C asymmetric oxygen bridges by breaking of the glycosidic bonds has significant implications for the structure of the CSP hydrogel. On one hand, it modifies the cross-linking pattern of the polymer network, contributing to a more stable structure that can withstand stress during the adsorption process. On the other hand, it affects the spatial arrangement of other functional groups, which may influence the accessibility of hydrophilic groups and, ultimately, the adsorption performance of the hydrogel for MB. In the CSP hydrogel, the stretching vibrations of hydroxyl groups are located at 3390 cm^−1^ and 3180 cm^−1^. The symmetric and asymmetric stretching vibrations of CH_2_ are at 2920 cm^−1^ and 2850 cm^−1^, respectively. The symmetric and asymmetric stretching vibrations of COO^−^ are at 1660 cm^−1^, 1550 cm^−1^, and 1410 cm^−1^. The C-N stretching vibration of the amide group is at 1240 cm^−1^. Among these, the carboxyl, hydroxyl, and amide groups are hydrophilic groups that swell upon water absorption in water, making them more conducive to the adsorption of methylene blue (MB). Simultaneously, the abundance of these organic functional groups in the CSP hydrogel material can provide more adsorption sites for the adsorption of MB organic dyes, making it an excellent organic adsorbent material. The absorption band observed in the CK sample within the range of 2070–2120 cm^−1^ can be attributed to the C≡C stretching vibrations of alkyne compounds present in the citrus sinensis peel. In the region below 1000 cm^−1^, which is typically referred to as the fingerprint region, the CK sample exhibits more complex vibrational features compared to CSP-1. This complexity is likely associated with the intricate skeletal vibrations of organic molecules derived from the Citrus sinensis peel.

### 3.3. Thermogravimetric Analysis

As depicted in Figure 4b, four distinct weight-loss stages can be clearly observed from the DTG curve of the CSP hydrogel, which are located at approximately 218, 339, 410, and 442 °C, with weight-loss rates of 5.11, 19.93, 3.91, and 3.42%, respectively. The weight loss in the first stage (around 218 °C) is primarily attributed to the loss of bound water within the hydrogel. It is worth noting that in our target application scenarios, such as farmland and water environments, where the temperature is typically below 100 °C, the mass loss of our CSP hydrogel is less than 5%, indicating its suitability for these applications. In the second stage (around 339 °C), the weight loss mainly results from the decomposition of the organic structure. Although we currently do not have specific data on the degradation range of Aam and AA units, we assume that within the temperature range of our interest (below 100 °C), these units remain relatively stable. For the third and fourth stages (around 410 °C and 442 °C, respectively), weight loss is mainly due to the release of some volatile substances during the combustion of carbon and other organic substances, such as carbonaceous materials and fructose. The thermogravimetric analysis reported above demonstrates that the decomposition-onset temperature of the CSP hydrogel exceeds 200 °C, which verifies its strong thermal stability.

### 3.4. Water-Absorption Properties of p(AA-co-Aam)-g-Citrus Sinensis Peel Hydrogel (CSP Hydrogel)

#### 3.4.1. Water-Absorption Curves

**Figure 5:**
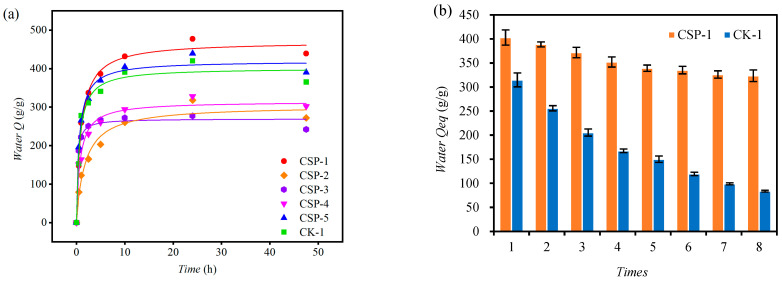
(**a**) Langmuir fitting curves of water absorption of hydrogels treated differently; (**b**) changes in the swelling ratio of CSP hydrogels during the water absorption–drying process. In this figure, CSP represents Citrus sinensis peel hydrogel, and CK-1 represents the hydrogel treatment without the addition of Citrus sinensis peel.

The water-absorption curves (Figure 5) indicate that the CSP-1 treatment exhibits the optimal water-absorption performance, with the highest water-absorption rate (Q) reaching 477 g/g within 24 h. In contrast, under the same reagent addition but without Citrus sinensis peel (CSP), the CK-1 treatment only attains 420 g/g. The Langmuir model can fit the water-absorption trends of these hydrogels well. As shown in Table 3, the correlation coefficients are all above 0.96. The theoretical maximum water-absorption rate of the CSP-1-treated hydrogel is 469.6 g/g, which is also higher than that of the CK-1 treatment (401.0 g/g). The k parameter in the Langmuir model represents the curvature of the curve, which, in this study, can indicate the rate at which the hydrogel reaches water-absorption equilibrium. A lower k value implies a faster rate. The k value required for the CK-1 treatment to reach equilibrium (0.634) is lower than that of the CSP-1 treatment (0.949), indicating that the CK-1 treatment reaches the equilibrium swelling rate in water more quickly. The observed trend in water absorption capacity (CSP-1 > CK-1 > CSP-3) is attributed to the interplay between MBA cross-linking density and CSP reinforcement, as elaborated in Section 4.2. Regarding the influence of the Aam:AA ratio on the swelling of the hydrogel when the amounts of CSP and MBA are constant, as further discussed in the Section 4, the amide groups from Aam and the hydroxyl groups from AA have different hydrophilicities, which jointly affect the swelling of the hydrogel. An appropriate adjustment of the Aam:AA ratio can optimize the water-absorption capacity of the hydrogel; this is also an important factor to consider in the preparation process of the hydrogel.

#### 3.4.2. Reusability of CSP Hydrogels

As can be seen from Figure 5b, after eight cycles of water absorption, the equilibrium swelling ratio (Water Qeq) of the CSP-1 treatment was 80.32% of that in the first water-absorption cycle. In contrast, for the CK-1 treatment without CSP addition, after eight cycles of water absorption, the water Qeq was only 26.62% of that in the first water-absorption cycle. This clearly shows that the addition of CSP can significantly enhance the reusability of hydrogels.

Since CSP-1 treatment had exhibited strongest performance, it was used as a CSP hydrogel for subsequent tests. The significant increase in the reusability of hydrogels with CSP addition is mainly attributed to the enhanced cross-linking stability provided by CSP. A more detailed explanation and supporting literature can be found in the Section 4.

#### 3.4.3. Water Absorption in Different pH Solutions

**Figure 6:**
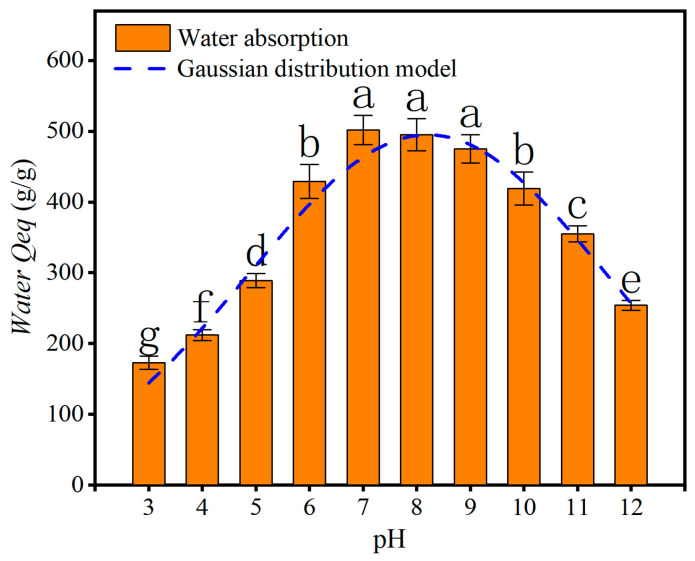
Water absorption of CSP hydrogel in different pH solutions. Different lowercase letters indicate significant differences at *p* < 0.05. The same applies hereafter.

The significant letter symbols in Figure 6 show that when the pH of the aqueous solution is 7–9, there is no significant difference in the equilibrium swelling ratio (Qeq) of the CSP hydrogel, and the water-absorption effect is the strongest. When the pH is 6 and 10, the water-absorption effect is second-best, followed by when the pH is 11. When the pH is 3, 4, 5, or 12, the Qeq of the CSP hydrogel is already lower than 300 g/g, which is far below the optimal Qeq at pH 7–9. Meanwhile, when we fit the graph using the Gaussian distribution model, a good fitting coefficient (R^2^ = 0.9661) is obtained, indicating that the equilibrium swelling ratio of the CSP hydrogel at different pH values follows a normal distribution. The Gaussian model is expressed as follows:

$$y=494.84\cdot e^{-\frac{{\left(x-8.205\right)}^{2}}{{2\times 3.316}^{2}}}$$

According to this formula, the maximum equilibrium swelling ratio (Qeq) of the hydrogel is 494.84 g/g, and the water-absorption magnification is the highest at pH = 8.205.

### 3.5. Water-Holding Properties of Different Soil Textures

**Figure 7:**
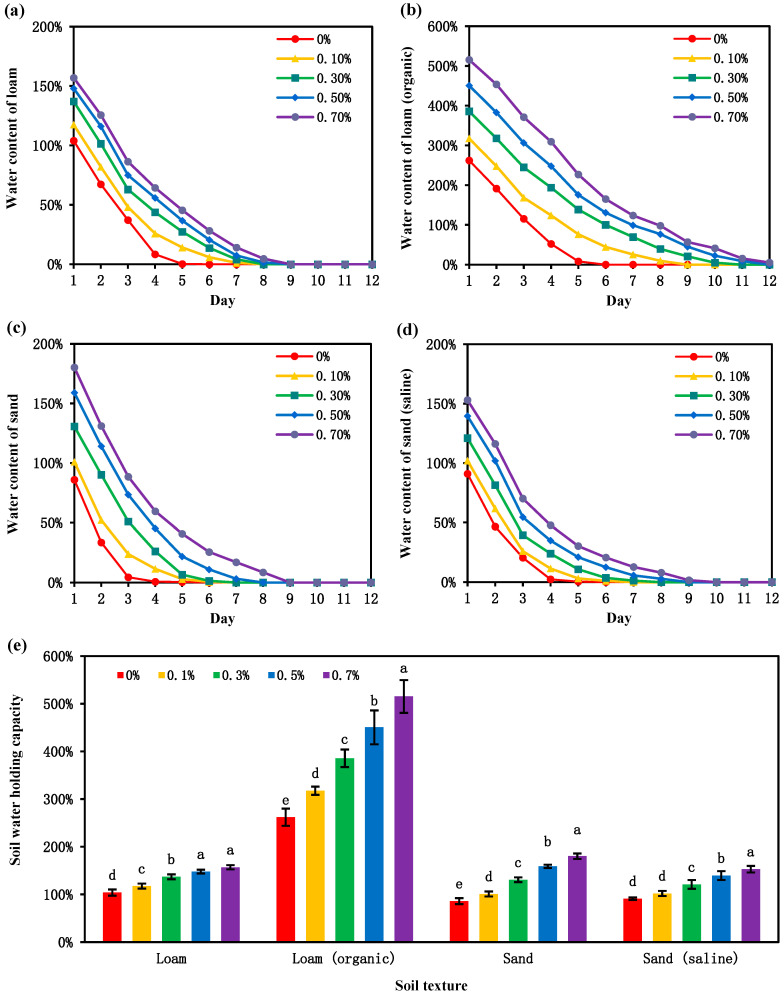
Soil moisture content curves of loam (**a**), sand (**b**), organic loam organic (**c**), and saline sand (**d**) with the addition of CSP hydrogel. (**e**) Influence of CSP hydrogel addition on soil moisture content on the first day. Different lowercase letters indicate significant differences at *p* < 0.05. The same applies hereafter.

In the northwest region, soils are mostly sandy soil and loam, with an uneven distribution of organic matter and a large amount of saline–alkali soil. Therefore, four types of soil—namely, loam, organic loam, sand, and saline sand—were used in this study. By comparing Figure 7a,c, it can be found that loam itself has a better water-absorption capacity than sandy soil. However, with the increase in the amount of CSP hydrogel added, the increase in the water-retention performance of sandy soil exceeds that of loam. As shown by comparing Figure 7a,b, when the organic matter content in loam increases, the soil bulk density decreases, increasing the expansion space for hydrogels, which can significantly improve the water-retention performance of hydrogels in organic loam. Comparing Figure 7c,d shows that when sandy soil is salinized, with the addition of hydrogels, the water-absorption effect is significantly lower than that of non-salinized sandy soil. From Figure 7e, it can be seen that the water-retention effect is the best in loam rich in organic matter, with an optimal 0.3% hydrogel addition amount. In loam, compared with the case without the addition of CSP hydrogel, 0.1%, 0.3%, 0.5%, and 0.7% additions increase the soil moisture content by 13%, 32%, 42%, and 51%, respectively. In organic loam, compared with the case without adding CSP hydrogel, 0.1%, 0.3%, 0.5%, and 0.7% additions increase the soil moisture content by 21%, 47%, 72%, and 97%, respectively. In sand, compared with the case without adding CSP hydrogel, 0.1%, 0.3%, 0.5%, and 0.7% additions increase the soil moisture content by 17%, 52%, 85%, and 110%, respectively. In saline sand, compared with the case without adding CSP hydrogel, 0.1%, 0.3%, 0.5%, and 0.7% additions increase the soil moisture content by 12%, 33%, 53%, and 68%, respectively.

### 3.6. Dye Adsorption Capacity and Durability of CSP Hydrogels

**Figure 8:**
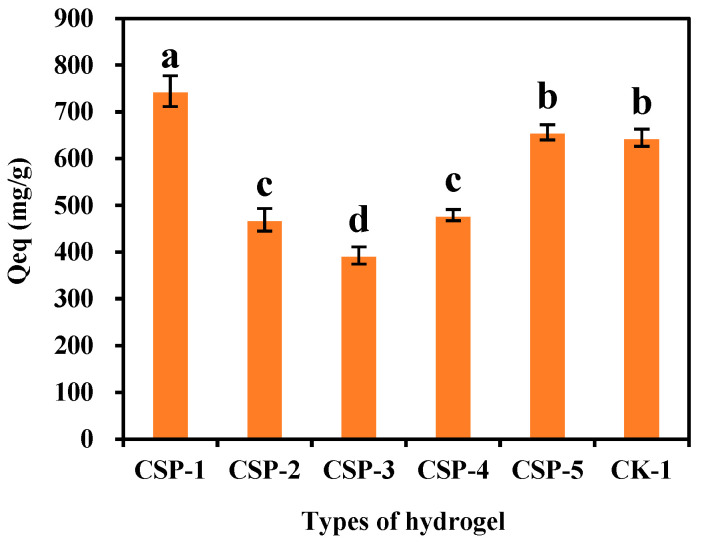
Adsorption of methylene blue by hydrogels with different treatments. In the figure, CSP refers to Citrus sinensis peel hydrogel, and CK refers to the hydrogel treatment without the addition of Citrus sinensis peel. Different lowercase letters indicate significant differences with *p* < 0.05. The same applies hereafter.

As depicted in Figure 8, the equilibrium adsorption capacity (Qeq) of CSP hydrogel-1 for MB solution was significantly higher than that of CSP hydrogel-2, CSP hydrogel-3, CSP hydrogel-4, and CSP hydrogel-5—by 58.59%, 89.45%, 55.36%, and 13.38%, respectively. It was also 15.41% higher than that of hydrogel-1 without the addition of Citrus sinensis peel (CSP). The remarkable superiority of CSP hydrogel-1 in terms of MB adsorption capacity can be primarily attributed to the role of CSP in forming a more stable three-dimensional structure as the skeleton of the hydrogel. In CSP hydrogel-1, CSP constructs a highly stable three-dimensional network with numerous and strong cross-linking points and interactions. Hydrogel-1, lacking CSP, cannot form such a stable three-dimensional skeleton. Without this stable framework, it fails to maintain its structure effectively in the presence of the MB solution, reducing the efficiency of MB molecule penetration and interaction with the adsorption sites. Additionally, the stable three-dimensional structure of CSP hydrogel-1 enables a more uniform distribution of functional groups, such as carboxyl, hydroxyl, and amide groups, which are essential for MB adsorption. In the less stable structures of other hydrogels, these functional groups may be less exposed or less organized, resulting in weaker interactions with MB molecules. Consequently, the CSP hydrogel-1 treatment was selected as the final treatment for CSP hydrogels in subsequent experiments for MB dye adsorption tests.

### 3.7. Influence of CSP Hydrogel Dosage on Dye Adsorption

**Figure 9:**
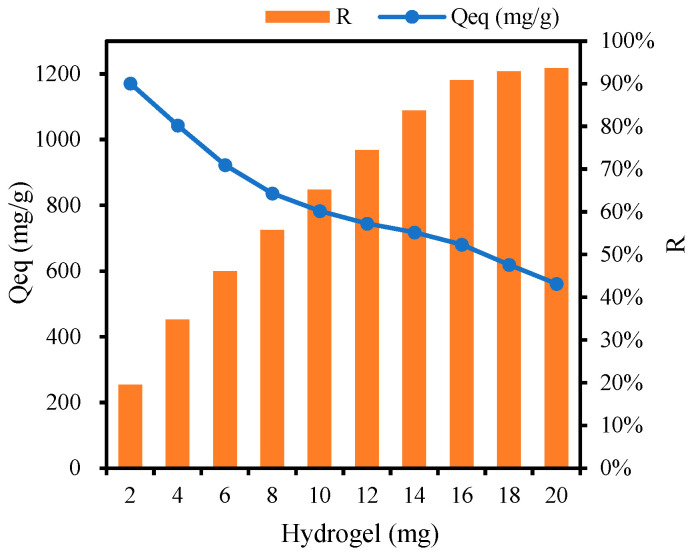
Influence of CSP hydrogel addition on Qeq and removal rate (R).

As presented in Figure 9, the adsorption of methylene blue (MB) significantly increases with increases in the dosage of hydrogel. Different amounts of CSP hydrogel were added to 25 mL of MB solution with a concentration (Ce) of 480 mg/L. When the dosages of hydrogel were 16, 18, and 20 mg, the adsorption rate (R) had already exceeded 90%, reaching 90.79%, 92.86%, and 93.61%, respectively, and tending towards stability. Meanwhile, the equilibrium adsorption capacities (Qeq) were 680.90, 619.06, and 561.67 mg/g, respectively.

### 3.8. Isothermal Adsorption Analysis

**Figure 10:**
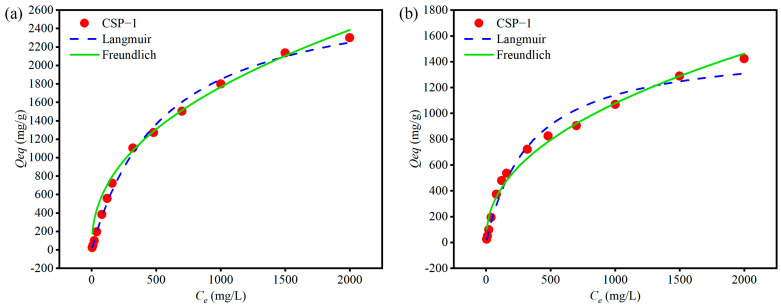

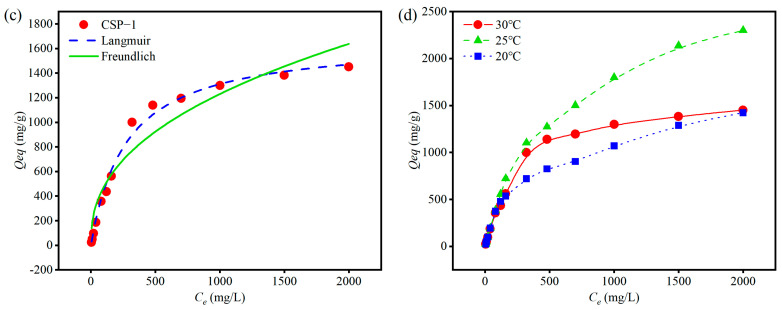
Langmuir and Freundlich models for the isothermal adsorption of methylene blue by CSP hydrogel at 25 °C (**a**), 20 °C (**b**), and 30 °C (**c**). (**d**) The influence of different temperatures on the adsorption effect of the hydrogel. In the figure, CSP-1 is CSP hydrogel.

To investigate the effect of CSP hydrogel addition on the adsorption capacity (Qeq) and removal rate (R), experiments were conducted by adding different masses of CSP hydrogel into solutions containing various initial concentrations (Ce) of methylene blue (MB) under controlled temperatures (20, 25, and 30 °C). The experimental data were fitted using Langmuir and Freundlich models, and the corresponding parameters are presented in Figure 10a–c, illustrating the adsorption isotherms at 25 °C. The Langmuir model provides the best fit with the highest R2 value compared to the Freundlich model, indicating that the Langmuir model better describes the monolayer adsorption behavior of CSP hydrogel on MB at 25 °C. Additionally, the Langmuir model better fits the data with higher R2 values than the Freundlich model at all three temperatures, suggesting that the adsorption process is more favorable at lower concentrations (Table 4). This is further supported by the trend in the n values obtained from Freundlich model fitting, which are highest at 25 °C, implying the stronger affinity of CSP hydrogel for MB at this temperature. From the comparison of adsorption isotherms at different temperatures in Figure 10d, it is evident that the optimal adsorption condition is not achieved at the highest temperature, as the adsorption efficiency at 25 °C surpasses that at both 30 °C and 20 °C. These findings collectively indicate that the CSP hydrogel exhibits an ideal balance between adsorption capacity and temperature, with the most efficient adsorption occurring at 25 °C. A detailed explanation regarding how the addition of CSP hydrogel affects the adsorption capacity at different controlled temperatures (20, 25, and 30 °C), as well as the reasons for the changes in the adsorption profile and ratio with increasing temperature, can be found in the Section 4.4.

### 3.9. Adsorption Kinetic Models

**Figure 11:**
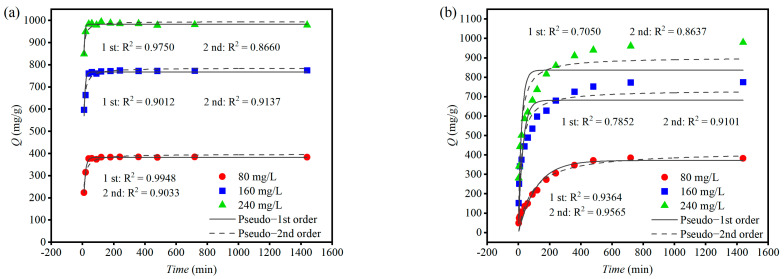
Adsorption kinetic tests of CSP hydrogel in MB solutions with different concentrations: (**a**) direct adsorption of hydrogel; (**b**) adsorption of hydrogel packed in tea bags.

Adsorption experiments were conducted by directly immersing 100 mg of CSP hydrogel into 500 mL of MB solution. As depicted in Figure 11a, rapid adsorption by the hydrogel was observed across different MB concentrations, achieving equilibrium adsorption within just 40 min. This indicates an exceptionally fast adsorption rate of MB onto CSP hydrogel. Pseudo first-order kinetic modeling provided a superior fit to the adsorption process, suggesting that the adsorption predominantly occurred during the initial stages and primarily involved physical adsorption mechanisms. Subsequent studies encapsulated the CSP hydrogel in tea bags under identical conditions, with the resulting adsorption dynamics illustrated in Figure 11b. It was observed that the adsorption rate slowed significantly, with higher concentrations taking longer to reach equilibrium adsorption. In this case, pseudo second-order kinetic modeling offered a better fit to the adsorption process, implying that confinement of the CSP hydrogel within tea bags shifted the adsorption mechanism towards a more integrated process throughout the duration of the experiment.

### 3.10. Desorption Effect of CSP Hydrogel on Methylene Blue

**Figure 12:**
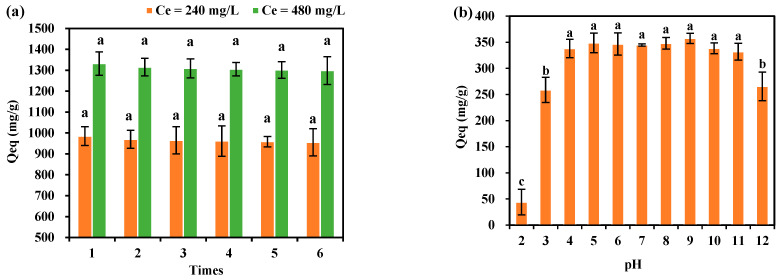
(**a**) Desorption of methylene blue by CSP hydrogel; (**b**) adsorption of methylene blue by CSP hydrogel at different pH values. Different lowercase letters represent a significant correlation between different times or pH.

The desorption capabilities of CSP hydrogel for different concentrations of methylene blue (MB) are presented in Figure 12. Twenty milligrams of CSP hydrogel were placed into 100 mL of MB. It can be observed that after multiple repeated desorptions in MB solutions with different concentrations, the Qeq of the CSP hydrogel did not change significantly. After six repeated desorptions in a 240 mg/L MB solution, the Qeq of the CSP hydrogel was 956 mg/kg, which is 97.05% of the initial value. After six repeated desorptions in a 480 mg/L MB solution, the Qeq of the CSP hydrogel was 1298 mg/kg, which is 97.45% of the initial value.

### 3.11. The Effect of CSP Hydrogel on the Adsorption of Methylene Blue at Different pH Values

Figure 12b shows the adsorption of methylene blue (MB) solutions by CSP hydrogel at different pH values. Five milligrams of CSP hydrogel were placed into 25 mL of 80 mg/L MB solutions with pH values ranging from 2 to 12. It can be seen that when the pH is in the range of 4–11, there are no significant differences in the adsorption of methylene blue (MB) by CSP hydrogel, and the Qeq is 331.87–357.46 mg/g. When the pH is 2, 3, or 12, the adsorption of MB by CSP hydrogel is significantly lower than that at pH values of 4–11. This indicates that CSP hydrogel has a relatively strong adsorption capacity in MB solutions within the pH range of 4–11.

## 4. Discussion

### 4.1. Characteristics of CSP Hydrogel

The Citrus sinensis peel (CSP) hydrogel exhibits a honeycomb-like 3D network under SEM (Figure 3b), contrasting sharply with the irregular porous structure of the control hydrogel (CK, Figure 3a). This morphological distinction stems from the graft copolymerization mechanism: fragmented CSP macromolecules act as a rigid backbone, cross-linking with pAA/pAAM chains to create a hierarchical porous system. The natural polysaccharide matrix restricts polymer chain aggregation during gelation, inducing phase separation that templates the honeycomb pattern. This honeycomb-like structure enables it to maintain relatively good stability, even after multiple uses (Figure 5b). Notably, Teng et al. [36] observed a wrinkled and uneven surface morphology in watermelon rind-derived hydrogels, which similarly contributed to enhanced mechanical robustness. While their study did not report a honeycomb-like architecture, both structural features—wrinkles (Teng et al.) and interconnected pores (in our work)—are indicative of hierarchically porous networks that improve stress dissipation. Importantly, Teng et al. demonstrated that their hydrogel exhibited significantly higher reusability (>90% swelling capacity after eight cycles) compared to conventional commercial hydrogels. Our material achieves comparable cyclic stability (80.32% swelling capacity after eight uses), further validating the utility of biomass-derived structural designs for durable hydrogel systems. Through FT-IR characterization, hydrophilic groups such as hydroxyl, carboxyl, and amide groups were identified in the hydrogel matrix. The porous surface structure observed via SEM (Figure 3b) facilitates exposure of these functional groups, enhancing interactions with water and organic dyes. These organic functional groups, including carboxyl, hydroxyl, and amide moieties, serve as hydrogen-bond donors and synergistically interact with the four hydrogen-bond acceptors present in the MB molecule [9]. This multi-site hydrogen-bonding mechanism significantly enhances the affinity and adsorption capacity of the hydrogel for MB, as demonstrated in previous studies on dye adsorption [38]. CSP contains a large amount of pectin, cellulose, and fructose, which are natural polymeric materials. When combined with polyacrylic acid and polyacrylamide, a more complex structure can be formed. This structure is less likely to decompose in water and has strong stability. Thermogravimetric analysis (TGA) results demonstrate that this material maintains excellent thermal stability, exhibiting minimal mass loss of less than 5% when heated below 100 °C, and can be used in relatively high-temperature environments.

### 4.2. Water Absorption by CSP Hydrogel

The CSP-1 treatment exhibits the most robust water-absorption performance, with an optimal component ratio of CSP:AA:AAM:MBA of 5:3:7:0.05. This observation underscores the critical role of MBA as a cross-linking agent, for which its concentration must be carefully balanced. Excessive MBA leads to an overly rigid hydrogel network, impairing water retention, whereas insufficient MBA weakens the three-dimensional structure, increasing solubility. This aligns with the findings of Zhang Zhibin et al. [39], who emphasized that hydrogels with low cross-linking densities achieve superior water absorption, further validating our experimental design. Notably, the water-absorption capacity initially decreases, then increases with higher AAM-to-AA ratios, suggesting that amide groups (from AAM) exhibit stronger hydrophilicity than hydroxyl groups (from AA). However, to mitigate risks associated with residual acrylamide toxicity, a conservative AA:AAM ratio of 7:3 was selected for final optimization. Importantly, the incorporation of Citrus sinensis peel (CSP) significantly enhances both water-absorption performance and reusability. This improvement likely stems from CSP-derived organic polymers (e.g., pectin and cellulose), which reinforce the hydrogel framework, as evidenced by similar strategies in graft copolymerization studies by Zhang Zhibin and Spagnol Cristiane et al. [39,40]. Their work demonstrated that oxidized macromolecules (e.g., starch and cellulose) synergize with hydrogels to surpass commercial products in water retention. Furthermore, the Gaussian distribution model reveals that the CSP hydrogel achieves peak water absorption at pH 8.2, favoring alkaline conditions. This phenomenon correlates with the ionization behavior of polyacrylic acid (PAA) within the hydrogel: H^+^ ions from PAA’s hydroxyl groups neutralize OH^−^ in alkaline solutions, enhancing swelling capacity. Consistent with this mechanism, Bingqin Teng et al. [36] reported that PAA-based hydrogels perform optimally in weakly alkaline environments. Collectively, these insights position CSP hydrogel as particularly advantageous for agricultural applications in alkaline soils, such as those prevalent in China’s northwest regions.

### 4.3. Improvement of Water Retention in Soils with Different Textures

The water-retention capacity of sandy soil (Sand) is inherently weaker than that of loam (Loam) due to its larger particle size and lower fractal dimension, which increase soil porosity and reduce water retention [41]. However, the addition of CSP hydrogels fundamentally alters this dynamic. By filling the interstitial pores in sandy soil, hydrogels facilitate structural expansion, thereby significantly improving water retention to levels surpassing that of untreated loam [42]. This phenomenon aligns with the findings of Mohawesh et al. [43], who emphasized the efficacy of hydrogels in coarse-textured soils. Interestingly, the interplay between soil organic matter and hydrogel performance reveals a contrasting trend in loam. When organic matter content increases, loam (organic) develops a lower bulk density and forms stable soil aggregates, achieving optimal intrinsic water retention. Concurrently, the reduced bulk density enhances the hydrogel’s swelling capacity, creating a synergistic effect. In saline environments, however, hydrogel performance is compromised. High salt ion concentrations in saline sandy soil inhibit hydrogel–water interactions, as monovalent cations (e.g., Na^+^) preferentially chelate with hydrogel polymer networks, reducing their osmotic swelling potential. This observation corroborates the work of Su Xiuxia et al. [44], in which it is mentioned that when hydrogels are in salt solutions, their water-absorption effect drops rapidly. This is because the chelating ability of different cations and anions plays an important role, and divalent cations are more likely to form stable chelates with hydrogels than monovalent cations. Moreover, the salt-absorption rate of hydrogels is affected by the radius of cations; larger cations have a lower charge density, resulting in a weaker shielding effect on anion groups. This increases the possibility of collision and reaction with anions, making it more difficult for them to penetrate the polymer network, thereby reducing the water-absorption rate.

### 4.4. Adsorption and Desorption of Methylene Blue (MB) by CSP Hydrogel

CSP hydrogel demonstrates robust methylene blue (MB) adsorption ability, which is primarily attributed to hydrogen-bond interactions between MB’s four acceptor sites and the hydrogel’s hydroxyl-rich polymer matrix (Figure 3a). This mechanism aligns with prior reports of hydrogen bond-dominated adsorption in polysaccharide-based hydrogels [45]. While increasing the CSP hydrogel dosage from 80 mg/L to 800 mg/L enhanced MB removal efficiency from 19.52% to 93.61%, the equilibrium adsorption capacity (Qeq) inversely decreased from 1171.21 mg/g to 561.67 mg/g (Figure 9), consistent with concentration-dependent active-site saturation phenomena observed in chitosan composites [46]. Isothermal analysis revealed superior fitting to the Langmuir model (R^2^ = 0.988) over the Freundlich model (R^2^ = 0.921), indicating monolayer adsorption dominance (Figure 10a–c). This contrasts with the multilayer adsorption patterns reported for bentonite–MB systems [47], highlighting CSP’s unique surface homogeneity. Notably, maximum adsorption occurred at 25 °C (Qeq = 2299.45 mg/g), which is contrary to the result in other studies that have suggested that the higher the temperature, the better the adsorption effect [48], decreasing by 36.93% at 30 °C (Figure 10d). Higher temperatures accelerate both the adsorption and desorption rates of CSP hydrogel, yet only an optimal temperature achieves the best adsorption result. This dual kinetic effect creates an optimal mid-range temperature window. Adsorption kinetics exhibited rapid equilibrium (<40 min) for freely dispersed hydrogel versus 180 min for tea bag-contained material (Figure 6). The 4.5-fold time difference quantitatively correlates with reduced surface accessibility, mirroring diffusion limitations reported for encapsulated activated carbon systems [49]. Despite this kinetic penalty, tea-bag containment achieved an equivalent ultimate Qeq (979 ± 32 mg/g), preserving practical utility for recoverable applications. pH robustness testing (Figure 12b) demonstrated a sustained Qeq >350 mg/g across pH values of 4–11, outperforming comparative materials such as acrylic acid-co-vinylsulfonic acid (optimal pH values of 7 and 9) [50] and hydrogel based on sodium styrene sulfonate (effective pH values of 7–9) [51]. It can be used as a potential MB-adsorbing material and play an important role in wastewater treatment and other fields.

After the CSP hydrogel adsorbed MB, multiple desorption tests using pure water revealed that the equilibrium adsorption capacity (Qeq) remained stable, at 97.05% and 97.45% of the initial value after six cycles. This retention highlights the hydrogel’s robust adsorption stability for MB, even after repeated desorption attempts. Notably, the strong interaction between hydrogen-bond donors (HBDs) and hydrogen-bond acceptors (HBAs) in the hydrogel–MB system likely underpins this stability, as evidenced by spectroscopic analyses in prior studies [52,53]. Importantly, this property positions CSP hydrogel as a promising candidate for MB-containing wastewater treatment, where long-term adsorption performance and minimal secondary pollutant release are critical [54]. However, the strong adsorption affinity poses challenges for material regeneration. While conventional aqueous washing fails to disrupt hydrogel–MB interactions, this limitation aligns with recent reports on hydrogen bond-dominated adsorbents [55]. To address this, future work should prioritize the optimization of solvent systems (e.g., pH-responsive or organic–aqueous mixtures) to achieve recyclability without compromising adsorption capacity. Ultimately, resolving this trade-off requires mechanistic studies correlating binding thermodynamics with hydrogel network dynamics, which will guide material redesign for broader environmental applications.

## 5. Conclusions

The incorporation of Citrus sinensis peel (CSP) significantly enhances water-absorption capacity (Qeq: 477g/g) and improves the reusability of the hydrogels. CSP hydrogel showed the best effect in terms of improving the water-retention property of organic loam. The optimal water-absorption performance of CSP hydrogels occurs at pH levels of 7–9, following a Gaussian distribution model. Compared to conventional hydrogels without CSP, these hydrogels demonstrate a stronger affinity for methylene blue (MB) and enhanced structural stability, reducing the likelihood of decomposition in water. According to the isothermal adsorption model, the CSP hydrogel shows the highest adsorption efficiency for MB at 25 °C, with an equilibrium adsorption capacity (Qeq) of 2299.45 mg/g under optimal conditions (25 °C, 12 h, and 2000 mg/L). Additionally, the pseudo first-order adsorption kinetic model provides a better fit when the hydrogel is directly used for MB adsorption. CSP hydrogel exhibits a robust adsorption capacity for MB across a wide pH range of 4–11 in aqueous solutions. These findings suggest that CSP hydrogels could be promising materials for water retention and pollutant removal in various environmental applications.

## Figures and Tables

**Table 1:** Reagent addition for CSP hydrogel synthesis under different treatments.

No.	CSP (wt%)	AAm (wt%)	AA (wt%)	MBA (wt%)	APS (wt%)
CSP-1	5	3	7	0.05	0.2
CSP-2	5	3	7	0.025	0.2
CSP-3	5	3	7	0.1	0.2
CSP-4	5	5	5	0.05	0.2
CSP-5	5	7	3	0.05	0.2
CK-1	0	3	7	0.05	0.2

**Table 2:** Test soil parameter table.

Soil Texture	Sand (%)	Silt (%)	Clay (%)	pH	SOC (g/kg)	Soil Capacity (g/cm^3^)	Soil Source
Loam	50	39	11	7.91	12.32	1.715	Collected in Lintan County, Gannan Tibetan Autonomous Prefecture, Gansu Province, China
Sand	92	1	7	8.16	0.74	1.628	Collected in Jinsha Village, Anning District, Lanzhou City, Gansu Province
Organic Loam	46	41	13	7.86	271.21	0.552	Procurement in Lanzhou City, Gansu Province
Saline Sand	88	11	1	8.94	7.03	1.586	Collected in Jiulongjiang Forest Farm, Ganzhou District, Zhangye City, Gansu Province

**Table 3:** Comparison table of Langmuir model parameters.

	q	k	R2
CSP-1	469.61	0.9492	0.9893
CSP-2	303.54	1.7738	0.9621
CSP-3	269.34	0.2099	0.9814
CSP-4	314.22	0.7596	0.9744
CSP-5	419.30	0.6030	0.9847
CK-1	401.03	0.6338	0.9669

**Table 4:** Statistical table of fitting parameters for Langmuir and Freundlich models.

	Langmuir (y = q × x/(k + x))			Freundlich (y = k × x^n)		
Temp (°C)	q	k	R^2^	n	k	R^2^
20	1531.9 ± 83.9	341.5 ± 55.5	0.9881	0.441 ± 0.024	51.2 ± 8.6	0.9837
25	2872.9 ± 89.3	554.3 ± 43.5	0.9956	0.434 ± 0.003	88.3 ± 1.9	0.9722
30	1672.9 ± 50.0	276.5 ± 26.2	0.9926	0.413 ± 0.049	70.7 ± 23.7	0.9301

## Data Availability

The original contributions presented in this study are included in the article. Further inquiries can be directed to the corresponding author.
